# Time optimal control‐based RF pulse design under gradient imperfections

**DOI:** 10.1002/mrm.27955

**Published:** 2019-08-23

**Authors:** Christoph S. Aigner, Armin Rund, Samy Abo Seada, Anthony N. Price, Joseph V. Hajnal, Shaihan J. Malik, Karl Kunisch, Rudolf Stollberger

**Affiliations:** ^1^ Institute of Medical Engineering Graz University of Technology Graz Austria; ^2^ Institute for Mathematics and Scientific Computing University of Graz Graz Austria; ^3^ School of Biomedical Engineering and Imaging Sciences King's College London London United Kingdom; ^4^ Johann Radon Institute for Computational and Applied Mathematics (RICAM) Austrian Academy of Sciences Linz Austria; ^5^ BioTechMed‐Graz Graz Austria

**Keywords:** gradient imperfections, gradient impulse response function, pulse design, simultaneous multi‐slice excitation, time optimal control

## Abstract

**Purpose:**

This study incorporates a gradient system imperfection model into an optimal control framework for radio frequency (RF) pulse design.

**Theory and Methods:**

The joint design of minimum‐time RF and slice selective gradient shapes is posed as an optimal control problem. Hardware limitations such as maximal amplitudes for RF and slice selective gradient or its slew rate are included as hard constraints to assure practical applicability of the optimized waveforms. In order to guarantee the performance of the optimized waveform with possible gradient system disturbances such as limited system bandwidth and eddy currents, a measured gradient impulse response function (GIRF) for a specific system is integrated into the optimization.

**Results:**

The method generates optimized RF and pre‐distorted slice selective gradient shapes for refocusing that are able to fully compensate the modeled imperfections of the gradient system under investigation. The results nearly regenerate the optimal results of an idealized gradient system. The numerical Bloch simulations are validated by phantom and in‐vivo experiments on 2 3T scanners.

**Conclusions:**

The presented design approach demonstrates the successful correction of gradient system imperfections within an optimal control framework for RF pulse design.

## 1 INTRODUCTION

MRI can be effectively accelerated by fast sequences^1^ or parallel imaging strategies in combination with advanced reconstruction techniques.^2^, ^3^ In addition to in‐plane parallel imaging, simultaneous encoding and acquisition of multiple slices (SMS) were shown to further increase the temporal efficiency of various clinically relevant MR sequences.^4^, ^5^

Basic SMS radio frequency (RF) pulses can be computed by a simple superposition of conventional single slice RF pulses with different carrier frequencies.^6^ This superposition, however, comes with the burden of a linear scaling of the maximal peak RF amplitude. To fulfill RF peak constraints, such SMS pulses are therefore typically stretched over long pulse durations. This can lead to conflicts with the achievable multiband (MB) factor, minimal echo time $T_{E}$, and echo spacing.^5^ The increased $T_{E}$ easily becomes a limiting issue for spin echo (SE) based applications such as turbo spin echo^7^ or high resolution diffusion imaging.^8^ Therefore, different RF pulse design methods have been introduced to reduce peak RF amplitudes^9^, ^10^, ^11^ or RF power by applying variable‐rate slice selective excitation (VERSE)^12^, ^13^, ^14^, ^15^, ^16^, ^17^ or power independent number of slices (PINS).^18^, ^19^

Alternatively, SMS RF pulses can be computed via optimal control.^20^, ^21^, ^22^ A refined optimal control model and method for the joint design of RF and slice selective gradient ($G_{s}$) shapes with the inclusion of all relevant hardware constraints as hard constraints was recently introduced.^21^ An extension of this optimal control method for the design of minimum duration pulses was presented in.^22^ These time optimal solutions not only fully exploit prescribed constraints on the designed waveforms such as RF and slice selective gradient peak amplitudes or slew rate limitations as well as slice profile and phase accuracy but also result in strongly time‐varying $G_{s}$ shapes.

Time‐variable gradients are known to be prone to gradient imperfections, including eddy currents with very long or short time constants, time delays, or bandwidth limited gradient amplifier that potentially limit their application. The linear time‐invariant gradient impulse response function (GIRF) has been shown to sufficiently assess the gradient performance^23^ for the correction of k‐space trajectory alterations in image reconstruction^24^ and pTx pulse design.^25^

In this work, we show that a direct inclusion of the GIRF in a time optimal control framework^22^ enables the design of short RF and $G_{s}$ shapes for SMS refocusing that inherently correct for measured gradient system imperfection. The proposed design method was tested via various SMS refocusing examples for a wide range of parameters. Additionally, phantom and in‐vivo data was acquired on 2 3T systems.

## 2 THEORY

This section contains the description of the GIRF, its impact on the numerical Bloch simulations as well as its inclusion in the time optimal control framework.^22^

### 2.1 Gradient impulse response function

Linear and time‐invariant (LTI) effects of the gradient system, including influences arising from the gradient amplifier, gradient coil as well as eddy currents and mechanical vibrations, can be characterized by the gradient impulse response function (GIRF).^23^

At this point, it is important to distinguish between the gradient amplitude $G_{s}$ that is included in the MR sequence, and its GIRF filtered version ${\tilde{G}}_{s}$ that is realized by the gradient system. Let *h*(*t*) be the GIRF that relates these 2 functions via a convolution in the time domain

(1) $${\tilde{G}}_{s}\left(t\right)=G_{s}\left(t\right)*h\left(t\right).$$

With known GIRF this equation allows an accurate prediction of the gradient field amplitude ${\tilde{G}}_{s}$ that is produced inside of the MR bore. Alternatively, the convolution operation can be applied in the frequency domain by multiplying the Fourier transform of the GIRF *H* with the Fourier transform of $G_{s}$

(2) $${\tilde{G}}_{s}=ℜ\left(F^{-1}HFG_{s}\right),$$

with H=F[h], the Fourier transform F and its inverse $F^{-1}$. In the discrete setting, $F^{-1},\;H,\;F$ are matrices whose dimensions and entries depend on the frequency resolution $\delta_{f}$ and bandwidth of *H* as well as the time resolution τ and pulse duration *T* of $G_{s}$. It should be noted that given by definition the LTI assumption does not account for non‐linear^26^ or time‐variable temperature dependences.^27^ We also restrict ourselves to the distortion on each gradient axis due to its own application, excluding cross terms, higher order spatial terms or $B_{0}$ variations.

### 2.2 Optimal control framework

The following optimal control framework is based on our preceding work,^21^, ^22^ which assumed an idealized gradient system model. We demonstrate how the inclusion of a refined GIRF‐based model can compensate for gradient imperfections and enhance the precision of the optimized pulses in practical applications. The key challenge for this is the inclusion of the GIRF into the optimal control model in order to fully profit from the optimization process. In particular, we pose the hardware constraints to the demand gradient waveform $G_{s}$ whereas the Bloch equation prediction and evaluation applies to the realized or GIRF filtered gradients ${\tilde{G}}_{s}$.

The numerical simulations are based on the spin domain Bloch equations with neglected relaxation terms. An uniform time grid is applied with $N_{t}$ time points for time *t*  ∈  [0, *T*] with a time step size $\tau \;=\;T/(N_{t}-1)$. A piecewise constant discretization for the complex‐valued RF, for the real‐valued $G_{s}$ and real‐valued ${\tilde{G}}_{s}$ is applied with values $B_{1,m}$, $G_{s,m}$, ${\tilde{G}}_{s,m}$ for $m\;=\;1,\;\ldots ,\;N_{t}-1$. In particular, Equation (2) is converted into a discretized matrix form ${\tilde{G}}_{s,m}\;=\;ℜ\left(F^{-1}HF\right)G_{s,m}$ and polar coordinates are used for $B_{1,m}\;=\;r_{m}\exp\left(i\vartheta_{m}\right)$ that allows direct description of the peak RF amplitude constraint with given matrices *F*, *H* specified in the experiments below.

The RF pulse design framework is completely general. However, since the focus of this work is to explore methods which include limitations in gradient performance, we limited the experimental design to real valued RF pulses and assumed an ideal RF hardware system.

The temporal evolution of the magnetization vector is solved by a series of complex rotation matrices based on the Cayley‐Klein parameters^28^, ^29^ $a_{m}$ and $b_{m}$

(3) $$a_{m}=\alpha_{m}a_{m-1}-\beta_{m}^{*}b_{m-1},$$

(4) $$b_{m}=\beta_{m}a_{m-1}+\alpha_{m}^{*}b_{m-1},$$

running from $m\;=\;1,\;\ldots ,\;N_{t}$ with the initialization $a_{0}\;=\;1$ and $b_{0}\;=\;1$ and coefficients

(5) $$\alpha_{m}=\cos\left(\phi_{m}/2\right)+i\gamma \tau z{\tilde{G}}_{s,m}\sin\left(\phi_{m}/2\right)/\phi_{m},$$

(6) $$\beta_{m}=i\gamma \tau B_{1,m}\sin\left(\phi_{m}/2\right)/\phi_{m},$$

(7) $$\phi_{m}=-\gamma \tau \sqrt{r_{m}^{2}+{\left(z{\tilde{G}}_{s,m}\right)}^{2}},$$

at spatial location *z* and the gyromagnetic ratio γ.

The prescribed magnitude and phase constraints are evaluated assuming a perfectly crushed SE refocusing profile $b_{N_{t}}{\left(z\right)}^{2}$ 29in a pointwise manner for a given field of view (FOV) in the slice direction consisting of $N_{z}$ spatial points *z*  ∈  [−*FOV*/2, *FOV*/2] using an equidistant spatial resolution $\delta \;=\;FOV/N_{z}$. Following^21^ the slice profile accuracy is modeled as small deviation from the ideal profile

(8) $$|b_{N_{t}}{|}^{2}\leq e_{\mathrm{out}}\left(z\right)\;\forall z\in \Omega_{\mathrm{out}},\;1-{\left|b_{N_{t}}\right|}^{2}\leq e_{\mathrm{in}}\left(z\right)\;\forall z\in \Omega_{\mathrm{in}},$$

where $\Omega_{\mathrm{out}}$ and $\Omega_{\mathrm{in}}$ are the domain parts out‐of‐slice and in‐slice, and $e_{\mathrm{out}},\;e_{\mathrm{in}}$ are their respective error bounds. Moreover, the phase spread inside each slice is desired to be nearly constant

(9) $$|\varphi -{\bar{\varphi }}_{l}|\leq e_{p}\left(z\right),\;\forall l=1,\;\cdots ,\;N_{s}$$

with mean phase ${\bar{\varphi }}_{l}$ of slice *l* out of the $N_{s}$ slices.

Besides slice profile constraints, the optimized RF pulses and gradient shapes need to fulfill technical constraints of the MR scanner hardware. Among these are amplitude constraints on the RF pulse, the pre‐emphasized $G_{s}$ shape and its slew rate $s_{m}$, which are essential to pass the hardware checks.

(10) $$0\leq r_{m}\leq r_{\text{max}},\;G_{s,m}\leq G_{\text{max}},\;\left|s_{m}\right|\leq s_{\text{max}},\;-\pi \leq \vartheta_{m}\leq \pi .$$

### 2.3 Optimal control method

The aim of the optimal control method is to optimize for the control $x\;=\;(r_{1},\;\cdots ,\;r_{N_{t}-1},\;\vartheta_{1},\;\ldots ,$ $\vartheta_{N_{t}-1},\;s_{1},\;\ldots ,\;s_{N_{t}-1})$ and the free terminal time *T* > 0 in order to minimize the cost function

(11) $$\begin{array}{cc} \underset{T>0,\;x}{\text{min}}J= & T+\frac{\tau \mu_{\mathrm{RF}}}{2}\sum_{\mathrm{time}}{\left|B_{1,m}\right|}_{2}^{2}+\frac{\tau \mu_{G}}{p}\sum_{\mathrm{time}}{\left(\frac{G_{s,m}}{G_{\text{max}}}\right)}^{p}+\frac{\delta \mu_{\mathrm{out}}}{2p}\sum_{\mathrm{outslice}}{\left(\frac{|b_{N_{t}}{|}^{2}}{e_{\mathrm{out}}}\right)}^{p}+\frac{\delta \mu_{\mathrm{in}}}{2p}\sum_{\mathrm{inslice}}{\left(\frac{|b_{N_{t}}{|}^{2}-1}{e_{\mathrm{in}}}\right)}^{p}\\ & +\frac{\delta \mu_{p}}{p}\sum_{\mathrm{slices}}\sum_{\mathrm{inslice}}{\left(\frac{\varphi -{\bar{\varphi }}_{l}}{e_{p}}\right)}^{p} \end{array}$$

subject to Equations ((3), (4), (5), (6), (7)) and

(12) $$0\leq r_{m}\leq r_{\text{max}},\;\left|s_{m}\right|\leq s_{\text{max}},\;-\pi \leq \vartheta_{m}\leq \pi ,$$

(13) $${\tilde{G}}_{s,m}=ℜ\left(F^{-1}HF\right)G_{s,m}.$$

Therein, the first 2 terms of Equation (11) constitute the time‐ and energy‐optimal cost function with weight $\mu_{\mathrm{RF}}\;>\;0$. The other terms are $L^{p}$‐penalization terms of the inequality constraints on the state variables where the exponent *p* is an positive even number (*P* > 2) that is increased throughout the optimization to achieve $L^{\infty }$ like error behavior. The regularization parameters $\mu_{\mathrm{RF}}$, $\mu_{G}$, $\mu_{\mathrm{out}}$, $\mu_{\mathrm{in}}$, $\mu_{p}\;>\;0$ are adapted after every 20th optimization step to ensure balanced penalty terms.^21^ This penalization method is needed here for coping with general inequality constraints. In contrast, the hardware limits on the controls Equation (12) can be included efficiently by projection‐based semismooth Newton/quasi‐Newton methods without increasing the computational effort or introducing additional parameters. For detailed background on the treatment of the inequality constraints in this context we refer to.^21^, ^22^

The maximal slice selective gradient amplitude $G_{\text{max}}$ and, the error bounds on the slice profile accuracy $e_{\mathrm{in}}$, $e_{\mathrm{out}}$ and the slice profile phase $e_{p}$ normalize the deviation to the desired state. For the sake of simplicity $e_{s}\;=\;\text{max}\left(e_{\mathrm{in}},\;e_{\mathrm{out}}\right)$ will be used later on to define the magnitude error of $e_{\mathrm{in}}$, $e_{\mathrm{out}}$. Additionally, we track the SAR estimate (W/kg)

(14) $$SAR_{e}={\text{SAR}}_{\mathrm{coileff}}\;f_{p}\tau \sum_{m=1}^{N_{t}}r_{m}^{2},$$

with a constant pulse rate $f_{p}$ (1/s) and coil efficiency ${\text{SAR}}_{\mathrm{coileff}}$ (W/kg/*μ* $T^{2}$)^8^ that impacts the weighting of $\mu_{\mathrm{RF}}$.

The optimization is done with the bilevel method for time optimal control as presented in our previous work.^22^ In particular, the lower level problem is solved for a fixed pulse duration employing adjoint‐based exact first derivatives and second derivatives supplied by a hybrid semismooth Newton/quasi‐Newton method with Broyden‐Fletcher‐Goldfarb‐Shanno (BFGS) update.^21^ To extend the bilevel method to the GIRF inclusion, the linear transformation Equation (13) is added prior to the Bloch simulation. According to the chain rule, this change is consistently transferred to the first derivatives.

## 3 METHODS

This section describes the implementation and details of the proposed pulse design and the performed experimental validation.

### 3.1 Experimental setup

The methods described in this paper have been tested using 2 separate Philips 3T MR systems (both Achieva). Scanner 1 has an experimental 8‐channel body coil^30^ with scan software capable of GIRF measurement using an image‐based procedure similar to.^31^ Scanner 2 is a standard clinical system with 2‐port birdcage transmitter with software implementation of SMS imaging techniques. Since neither system had both capabilities, SMS imaging was performed by scanner 2 using pulses corrected for the GIRF measured from scanner 1. Though not ideal, prior experience suggests that the gradient performance of both systems is similar^17^; errors that may arise from assuming this equivalence will be discussed later.

**Figure 1:**
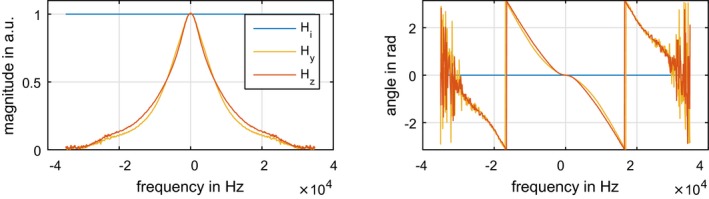
Magnitude and phase angle of the idealized ($H_{i}$) and measured GIRF in y ($H_{y}$) and *z*‐direction ($H_{z}$)

Figure 1 depicts the idealized ($H_{i}$) and the measured transfer functions along the y ($H_{y}$) and z ($H_{z}$) direction with frequency resolution $\delta_{f}\;=\;76.3$ Hz. The 2 measured GIRFs both have low‐pass characteristics but differ in cutoff frequency (4200 Hz for $H_{y}$ and 3750 Hz for $H_{z}$) where the magnitude response is reduced by a factor of $1/\sqrt{2}$. Moreover, the phase deviation potentially leads to a varying phase shift of different frequency components. The GIRF data and details about its application are publicly available (https://github.com/mriphysics/reVERSE-GIRF).

### 3.2 Pulse design

The presented optimization approach is an extension of our preceding work^22^ that can be downloaded from https://github.com/rundar/mr.control. The algorithm was implemented in MATLAB (The MathWorks, Inc., Natick) with pre‐compiled C‐based MEX files for an accelerated parallel solution (OpenMP) of all Bloch simulations and derivative computations. All calculations were done in parallel on the high‐performance computing cluster “RADON 1” (RICAM, Linz, Austria) using 1 node (2x Xeon E5‐2630v3 with in total 16 cores and 128 GB of RAM) for each case. All shown examples were designed for SMS refocusing assuming perfect spoiling with varying multiband factor (MB), time‐bandwidth‐product (TBWP), slice‐thickness (THK) and field of view (FOV). The global head SAR estimate $SAR_{e}$, see Equation (14), was computed with a fixed pulse frequency $f_{p}\;=\;16.67$ 1/s and SAR efficiency ${\text{SAR}}_{\mathrm{coileff}}\;=\;0.25$ W/kg/μ$T^{2}$ for a representative 3T birdcage coil.^8^ As testing was performed on the brain (see below), we adopted the head SAR limit of ${\text{SAR}}_{\text{max}}\;=\;3.2$ W/kg.

The hardware constraints on the amplitudes of the RF $r_{\text{max}}\;=\;13$ *μ*T, slice selective gradient $G_{\text{max}}\;=\;30$ mT/m and its slew rate $s_{\text{max}}\;=\;180$ T/m/s were chosen to comply with the experiments on Philips Achieva 3T MR systems with maximal hardware limits of 40 mT/m and 200 T/m/s (Philips Healthcare, Best, Netherlands). Additionally, the time discretization of the RF and $G_{s}$ shapes was set to the minimal gradient raster time τ = 6.4 μs of the previously mentioned MR systems and remains constant throughout the optimization. The iterative bilevel optimization was always initialized according to our preceding work,^22^ with PINS^18^ RF and $G_{s}$ shapes based on a SLR^29^ sub‐pulse assuming a perfectly crushed SE with the parameters $d_{1}\;=\;0.01/4$ and $d_{2}=0.01/\sqrt{2}$. The resulting initial pulse durations ranged from *T* = 7.48 ms (MB = 3, TBWP = 2, THK = 2 mm and FOV = 120 mm) to *T* = 24.67 ms (MB = 5, TBWP = 4, THK = 1 mm and FOV = 120 mm). The error bounds of the deviation from the ideal refocusing profile were set to $e_{s}\;=\;1-3$%.

One set of parameters (MB = 4, TBWP = 4, THK = 2 mm and FOV = 120 mm) was analyzed and compared in more detail to examine the impact of an idealized and measured GIRF on the proposed pulse design. For this purpose, we optimized 2 scenarios using the ideal unit response ($H_{i}$) and the measured GIRF along the *z*‐direction ($H_{z}$). Both optimization runs were initialized with the same PINS pulse. The 3 pulse candidates were evaluated and, following GIRF application ($H_{i}$ and $H_{z}$), were additionally compared with Bloch simulations. To examine the effect of time‐invariant $B_{0}$ and $B_{1}$ inhomogeneities on the optimized RF and ${\tilde{G}}_{s}$ shapes we computed Bloch simulations with a $B_{1}$ scaling of 75‐125% $B_{1}$ and an off‐resonance range of $\Delta B_{0}\;=\;\pm \;200$ Hz. The impact of $\Delta B_{1}$ and $\Delta B_{0}$ on the refocusing profile was analyzed for 2 representative slices.

A second set of parameters (MB = 5, TBWP = 4, THK = 2 mm and FOV = 120 mm) was analyzed with different assumptions regarding phase constraints and using different GIRFs in pulse design and evaluation. Firstly, optimization was performed with and without constraints on the phase angle of the refocusing profile. For the former, the maximum allowed deviation of the phase angle from the mean phase was $e_{s}\;=\;0.25$ rad per slice. In all other optimization scenarios, unless stated otherwise, explicit phase angle constraints were included to reduce the phase spread of the refocusing profiles. Secondly, we tested the effect of assuming an ideal gradient system ($H_{i}$). We then evaluated the optimized pulse and $G_{s}$ shape with measured GIRFs of different gradient directions ($H_{z}$, $H_{y}$ and $H_{\mathrm{yz}}$) with $H_{\mathrm{yz}}$ being the arithmetic mean of $H_{z}$ and $H_{y}$. Thirdly, we analyzed the impact of using different gradient axes in optimization and evaluation ($H_{z}$, $H_{y}$ and $H_{\mathrm{yz}}$).

To test the influence of TBWP, MB, THK, and FOV on the proposed optimization method, pulses were computed for different parameter combinations (MB = 3‐8, TBWP = 2‐4, THK = 1‐5 mm and FOV = 90‐210 mm). The optimization was done with phase angle constraints for the ideal unit response ($H_{i}$) and measured GIRF ($H_{z}$).

### 3.3 Experimental validation

The numerical simulations were validated by phantom measurements using an 8‐channel head coil on scanner 1. Using 3 MB factors (MB = 3, 4 and 5), phantom measurements were performed to measure the slice profile of a crushed SE sequence. All other parameters (TBWP = 4, THK = 2 mm, FOV = 120 mm, $e_{s}\;=\;1$% and $e_{p}\;=\;0.025$ rad) were identical. For each MB factor 2 sequences were created using $H_{i}$ and $H_{z}$ optimized results. For excitation we computed SLR^29^ based phase optimized superposition MB pulses with matching slice profiles.^32^ The measurements of the slice profile were performed along the *z*‐direction (transversal) for a homogeneous bottle phantom filled with mineral oil. The sequence parameters were set to $T_{E}/T_{R}\;=\;16/100$ ms, FOV = 140 × 140 mm, in‐plane resolution = 0.5 × 0.5 mm, matrix = 1024 × 1024). The slice profile measurements were corrected by an intensity profile of a sagittal single slice GRE reference measurement (THK = 2 mm) with the same sequence parameters.

In vivo scans of a healthy male volunteer were conducted using the same 8‐channel head coil on scanner 2.^17^ Two optimized $H_{i}$ and $H_{z}$ RF pulses (MB = 4, TBWP = 4, THK = 2 mm, FOV = 120 mm, $e_{s}\;=\;1$%, $e_{s}\;=\;0.025$ rad) were scaled down by a factor of 9 to be used as low tip angle excitation pulses. The scaling was carried out only for the purpose of experimental demonstration. The optimized slice selective gradient shapes were adopted without alterations. Two GRE sequences with blipped‐CAIPI shift were created with $T_{E}/T_{R}\;=\;11/200$ ms, FOV = 240 × 240 mm, in‐plane resolution = 0.75 × 0.75 mm, matrix = 480 × 480, CAIPI shift = 3. The aliased MB data were reconstructed with a SENSE‐based algorithm using ReconFrame (GyroTools GmbH, Zürich, Switzerland).

## 4 RESULTS

This section demonstrates the compensation of gradient system imperfections for time optimal SMS refocusing with highly varying $G_{s}$ shapes with numerical simulations and experimental phantom and in vivo measurements on a 3T MR system.

### 4.1 Simulations

**Figure 2:**
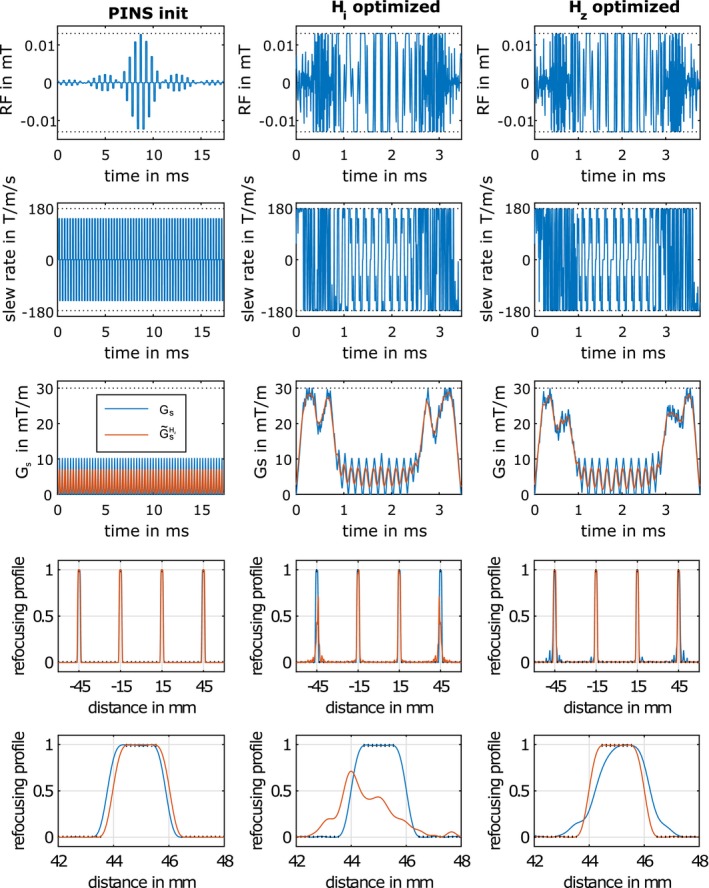
Impact of the measured GIRF $H_{z}$, see Figure 1, on 3 different slice selective gradient shapes and simulated refocusing profiles. Column 1 depicts the PINS initial used as an educated guess to initialize the $H_{i}$ (column 2) and $H_{z}$ (column 3) optimization (with constraints on the phase angle of the refocusing profile). Row 1 depicts the RF pulse and row 2 depicts the slew rate of the slice selective gradient. Row 3 depicts the slice selective gradient shapes before ($G_{s}$ in blue) and after convolution with the $H_{z}$ GIRF (${{\tilde{G}}_{s}}^{H_{z}}$ in orange) together with the magnitude constraints (dotted black). Rows 4 and 5 show the simulated refocusing profiles $|b_{N_{t}}{\left(z\right)|}^{2}$ before (blue) and after convolution with the $H_{z}$ GIRF (orange) together with the spatial magnitude constraints (dotted black). Note that in the center column the blue curve performs as expected and the orange curve shows substantial damage. In the third column the reverse is the case, with the orange curve following the desired performance

The 3 columns in Figure 2 correspond to the PINS initial, $H_{i}$ and $H_{z}$ optimized results. Descending the RF and slew rate of $G_{s}$ together with $G_{s}$ and the filtered ${{\tilde{G}}_{s}}^{H_{z}}$ shapes and the corresponding simulated refocusing profiles (MB = 4, TBWP = 4, THK = 2 mm and FOV = 120 mm) are displayed. The overall duration of the optimized results reduced from 17.41 ms (PINS initial) to 3.46 ms (optimized assuming an ideal uniform response $H_{i}$) and 3.78 ms (optimized with the measured GIRF along the *z*‐direction $H_{z}$). The point‐wise constraints on the RF and $G_{s}$ amplitudes as well as on the refocusing profile are depicted by black dotted lines. It can be seen that the optimized RF and $G_{s}$ waveforms often attain the bound constraints for the control variables (RF amplitude and $G_{s}$ slew rate). Although not shown, the optimization runs were performed with explicit constraints on the phase spread of the refocusing profiles ($e_{p}\;=\;0.025$ rad). The simulated refocusing profiles for the $G_{s}$ and ${{\tilde{G}}_{s}}^{H_{z}}$ shapes of the PINS initial resulted in a slice shift of 0.2 mm for the outermost slice, see bottom row of Figure 2. In contrast, the impact of $H_{z}$ on the $G_{s}$ waveform was considerably more pronounced for the optimized cases. The fourth row depicts the slice profiles either without filtering (blue curve, using $H_{i}$) or with realistic $H_{z}$ filtering (orange curve). The classical optimized case (column 2) results in distorted refocusing profiles for ${{\tilde{G}}_{s}}^{H_{z}}$ (orange curve). This is most noticeable in row 5, which displays a magnified view of the outermost slice. Inclusion of $H_{z}$ in the optimization led to complete compensation of slice profile degeneration, as seen in column 3, where the simulated refocusing profile (orange line) precisely fulfills the prescribed constraints on the refocusing profile.

**Figure 3:**
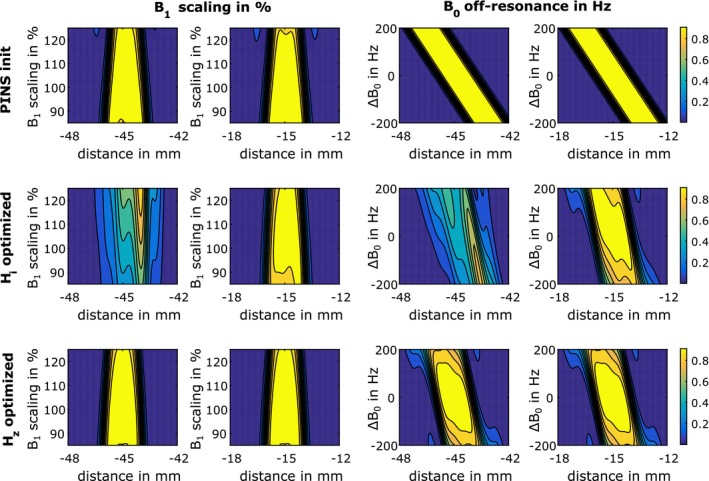
Simulated refocusing profiles $|b_{N_{t}}{\left(z\right)|}^{2}$ for a variation in the $B_{0}$ off‐resonance and $B_{1}$ inhomogeneity using the $H_{z}$ filtered slice selective gradient shapes ${{\tilde{G}}_{s}}^{H_{z}}$ shown in Figure 2. Columns 1 and 3 show a magnified view of to the outermost slice and columns 2 and 4 show a magnified view to a central slice. The other 2 slices result in similar refocusing patterns

Figure 3 depicts refocusing profiles for a $B_{1}$ variation of 85‐125% (column 1 and 2) and $B_{0}$ offset range of ±200 Hz (column 3 and 4) for 2 representative slices. The other 2 slices showed similar results. The simulations were performed with the ${{\tilde{G}}_{s}}^{H_{z}}$ shapes shown in Figure 2. The inclusion of the realistic $H_{z}$ filtering in the last row improves the slice profiles over the entire simulated $B_{0}/B_{1}$ range, especially for the outermost slice.

**Figure 4:**
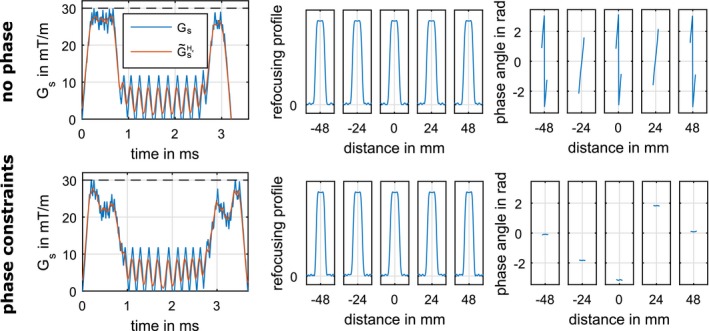
Comparison of 2 $H_{z}$ GIRF optimized MB = 5 examples with and without a distinct constraint on the phase spread of the refocusing profile with otherwise identical parameters. Column 2 shows an enlargement of 6 mm to demonstrate the simulated refocusing profiles $|b_{N_{t}}{\left(z\right)|}^{2}$ and Column 3 shows an enlargement of 6 mm to see the phase spread $\arg(b_{N_{t}}{\left(z\right)}^{2})$ of each slice

Figure 4 compares the impact of constrained phase angles of the refocusing profiles for 1 representative example (MB = 5, TBWP = 4, THK = 2 mm, FOV = 120 mm, $e_{s}\;=\;2$%). Row 1 shows the $H_{z}$ optimized result without phase angle constraints and row 2 shows the $H_{z}$ optimized result with an allowed phase deviation $e_{p}\;=\;0.025$ rad. There are only minor differences in the magnitude of the refocusing profiles (column 2). The consideration of the phase angle, however, resulted in a much less pronounced phase deviation with slightly longer pulse durations (3.69 ms compared to 3.58 ms without a phase angle constraint).

**Figure 5:**
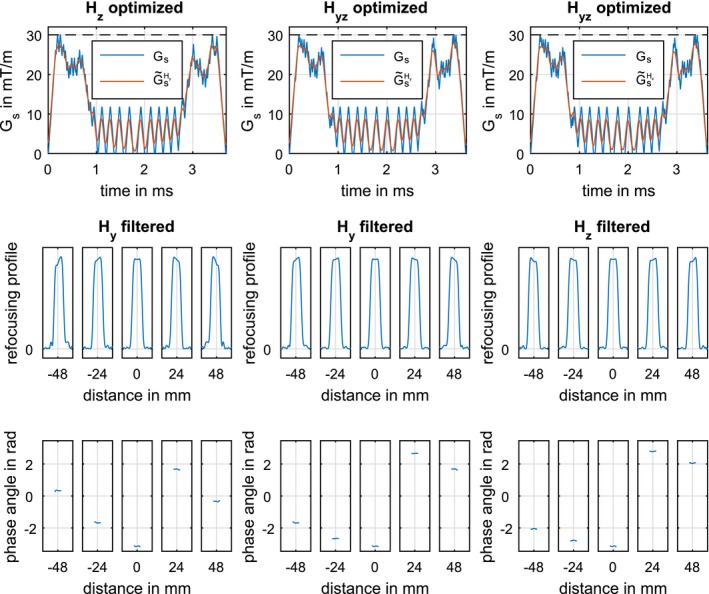
Comparison of 2 $H_{z}$ and $H_{\mathrm{yz}}$ optimized MB = 5 examples filtered for different GIRF directions before Bloch simulation. Column 1 shows the $H_{y}$ filtered result for the $H_{z}$ optimized example shown in Figure 5. Columns 2 and 3 show the $H_{y}$ and $H_{z}$ filtered result for the $H_{\mathrm{yz}}$ (average of $H_{y}$ and $H_{z}$) optimized example. Row 2 shows an enlargement of 6 mm to demonstrate the simulated refocusing profiles $|b_{N_{t}}{\left(z\right)|}^{2}$ and Column 3 shows an enlargement of 6 mm to see the phase spread $\arg(b_{N_{t}}{\left(z\right)}^{2})$ of each slice

Next, we investigated the impact of different slice orientations. Figure 5 shows the $H_{z}$ optimized example of Figure 4 filtered with $H_{y}$ (column 1) and an optimized example using an averaged transfer function ($H_{\mathrm{yz}}$) filtered with $H_{y}$ (column 2) and $H_{z}$ (column 3). The appearance of the different gradient shapes was similar across all optimized results. Regarding the different gradient directions, only small changes were observed for the simulated slice profiles. Again, the differences were less pronounced for the central slices.

Table 1 summarizes the performance of optimized pulse candidates (MB = 5) for different gradient directions with and without explicit phase constraints for different GIRF directions. The table compares maximal slice ($e_{s}$) and phase errors ($e_{p}$) of the refocusing profile using the $H_{z}$ filtered slice selective gradient ${{\tilde{G}}_{s}}^{H_{z}}$, SAR estimates $SAR_{e}$ and the pulse duration *T*. The maximal refocusing profile deviation increases, compared to the 2% refocusing error constraint used in the optimization, to roughly 18% for the $H_{y}$ optimized case evaluated with the $H_{z}$ GIRF. The use of an averaged GIRF $H_{\mathrm{yz}}$ in the optimization results in an intermediate case with a low slice profile error for both gradient directions. The evaluation of the refocusing accuracy with respect to $H_{y}$ and $H_{\mathrm{yz}}$ is summarized in Supporting Information Table S1.

**Table 1:** Performance of initial and optimized SMS refocusing pulses (MB = 5, TBWP = 4, THK = 2 mm and FOV = 120 mm) for different GIRF directions ($H_{y}$, $H_{z}$ and $H_{\mathrm{yz}}$) with and without constraints on the phase angle of the refocusing profiles

Initial	GIRF	Max $\left|e_{s}\right|$ a.u.	Max $\left|e_{p}\right|$ rad	$SAR_{e}$ W/kg	*T* ms
		0.010	0.015	0.61	16.14
Without phase constraints	$H_{i}$ optimized	0.944	1.108	1.84	3.29
	$H_{y}$ optimized	0.182	1.539	1.98	3.76
	$H_{z}$ optimized	0.020	2.263	1.93	3.58
	$H_{\mathrm{yz}}$ optimized	0.087	0.746	1.86	3.60
With phase constraints	$H_{i}$ optimized	0.923	0.675	1.65	3.42
	$H_{y}$ optimized	0.138	0.053	1.82	3.64
	$H_{z}$ optimized	0.020	0.024	1.86	3.69
	$H_{\mathrm{yz}}$ optimized	0.069	0.031	1.89	3.63

All pulses are evaluated using the $H_{z}$ GIRF filtered slice selective gradient ${{\tilde{G}}_{s}}^{H_{z}}$ to analyze the influence of the GIRF direction. Depicted are the GIRF used in the optimization, maximal refocusing profile ($e_{s}$) and phase ($e_{p}$) errors, the SAR estimate ($SAR_{e}$) and the overall pulse duration (*T*). Examples of this table are also used in Figures 4 and 5 for further analysis.

**Figure 6:**
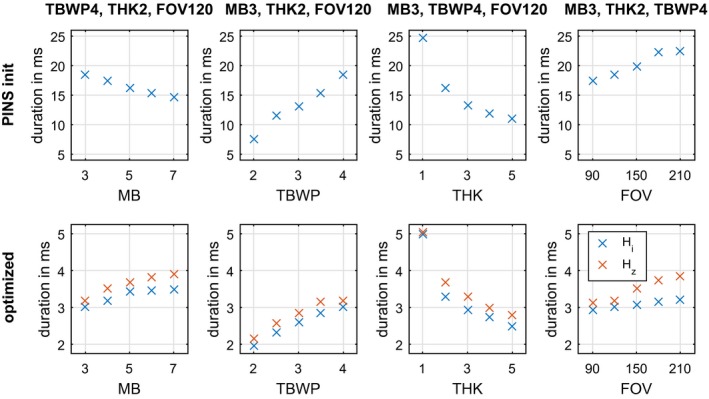
Overview of the initial PINS and the $H_{i}$ and $H_{z}$ optimized pulse durations for a variation of different parameters including time bandwidth product (TBWP), multiband factor (MB), slice thickness (THK), and field of view (FOV). A more detailed overview and description of the different examples is given in Supporting Information Figures S1‐S4 and Supporting Information Table S2

Finally, we investigated the influence of different parameters (TBWP, MB, THK, and FOV) on the proposed GIRF‐corrected pulse design method. The optimization robustly computed short pulse candidates that fulfilled all prescribed constraints. The performance of all $H_{i}$ and $H_{z}$ optimized results are summarized in Figure 6 showing the optimized pulse durations in comparison with the PINS pulse durations. More details of all optimized examples are given in Supporting Information Table S2 and Supporting Information Figures S1‐S4. For the sake of clarity, the following results were documented for the $H_{z}$ optimized results only since the $H_{i}$ optimized results behave similarly. The pulse duration of the optimized results ranged from 3.19 ms to 3.90 ms (MB = 3‐7), 2.16 ms to 3.19 ms (TBWP = 2‐4), 2.78 ms to 5.04 ms (THK = 1‐5 mm) and 3.12 ms to 3.84 ms (FOV = 90‐210 mm). Supporting Information Figures S1‐S4 depict 3 optimized results for each parameter variation. The average computation time of all $H_{i}$ and $H_{z}$ optimized pulses is approximately 30 minutes on the hardware described above.

### 4.2 Experiments

**Figure 7:**
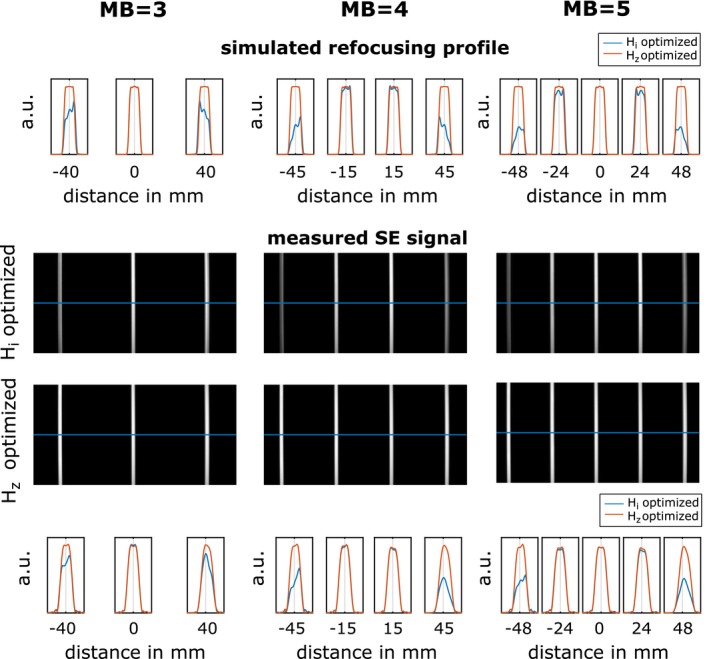
Simulated and measured SE phantom data of $H_{i}$ (blue) and $H_{z}$ (orange) optimized MB = 3, MB = 4, see Figure 2, and MB = 5 results on scanner 1. The first and last row shows an enlargement of 6 mm of the simulated and measured slice profiles

Figure 7 shows the simulated and experimentally measured SE profiles on scanner 1 for 3 $H_{i}$ and $H_{z}$ optimized refocusing pulses with varying MB factor (3, 4 and 5). Results related to MB = 4 are also shown in Figure 2. The measured slice profile of all experiments matched simulated results and showed the expected slice profile distortions for the $H_{i}$ optimized candidates. These distortions are corrected in the case of $H_{z}$ optimization.

**Figure 8:**
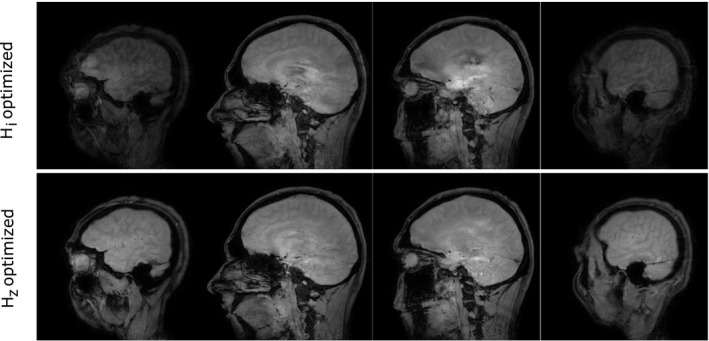
Reconstructed in vivo GRE acquisitions on scanner 2 using scaled MB = 4 RF pulses, see Figure 2, to achieve a nominal flip angle of 20∘. The $H_{i}$ optimized RF pulses result in distorted slice profiles of the outer slices, which lead to a reduction in acquired signal. The $H_{z}$ optimized RF pulses correct for this and consistent signal is achieved in all 4 slices

Figure 8 shows acquired in vivo gradient echo (GRE) images. As previously mentioned, the $H_{i}$ and $H_{z}$ optimized RF pulses were scaled to enable their use as low tip excitation pulses and to perform the scans on a scanner 2 with a GIRF not identical but close to $H_{z}$. Supporting Information Figure S5 shows the expected performance of the 9x‐downscaled RF pulses in the excitation regime using the GIRF of scanner 1. The simulated excitation profiles were evaluated in terms of the flip angle $\sin^{-1}(|2a_{N_{t}}\left(z\right)b_{N_{t}}{\left(z\right)}^{*}\left|\right)$. The inclusion of the GIRF compensates slice profile distortions and results in clean slice profiles with a flip angle of $20^{\circ }$. Compared to Figure 2 there is an increased but still moderate error below 3%. The predicted signal recovery of the outer slices is in good accordance with the observed in vivo results. The reconstructed and separated in‐vivo images on scanner 2 show a clear signal reduction for the outer slices, which is compensated using the $H_{z}$ optimized waveforms. Furthermore, the $H_{z}$ optimized waveforms have the advantage of reduced residual slices outside the field of view (not shown). The residual slices of the $H_{i}$ optimized pulses resulted in signal crosstalk, as displayed in the center of the third slice (Figure 8). In contrast, the use of $H_{z}$ optimized waveforms results in the desired consistent signal intensity.

## 5 DISCUSSION

The inclusion of system specific hardware and safety constraints allows direct design of short SMS refocusing pulses to reduce $T_{E}$ or echo spacing of existing clinically relevant SE sequences.^8^ The achieved accuracy of highly fluctuating time optimal slice selective gradient shapes further depends on the performance of the gradient system and leads to the desired results only for gradient systems with a sufficiently high bandwidth.^22^ In this work, we therefore presented an extension of the optimal control framework^21^, ^22^ to correct for spatially linear gradient system imperfections in the pulse design. The inclusion of a measured GIRF^23^ in the optimization framework resulted in pre‐emphasized $G_{s}$ shapes that compensate for gradient system imperfections and that can be implemented without additional post‐processing. The proposed framework is flexible in the optimization goal and allows for a tailored balance between a short pulse duration, small slice profile deviations, and RF power requirements. The presented GIRF‐corrected SMS pulse design method robustly delivered short refocusing pulses for a variety of design parameters (MB factors, TBWP, THK, and FOV).

The various SMS refocusing cases presented in this work are typical configurations where the minimal pulse duration is limited by peak RF, $G_{s}$ and slew rate amplitudes as well as the overall RF power. The hardware constraints and the measured GIRF chosen matched the 3T MR system used for experimental validation. These constraint values are input parameters and can be adapted to match different MR hardware. To achieve the best results with respect to the minimal pulse duration and refocusing accuracy it is therefore highly recommended to use the vendor specific hardware limitations and a GIRF that describes the gradient system and direction used.

The GIRF model predicts the actual slice selective gradient shape ${\tilde{G}}_{s}$ generated inside of the MR scanner's bore for a given demanded $G_{s}$ shape.^23^ In contrast to an idealized GIRF $H_{i}$, the low‐pass character of the measured $H_{y}$ and $H_{z}$, see Figure 1, suppresses higher frequency components and results in smoothed $G_{s}$ shapes. It is important to mention that the GIRF models the gradient transfer function of a specific scanner model and gradient direction. Therefore, the GIRF varies for different gradient directions and substantially between models and vendors. Additional phase delays further result in temporal mismatches between RF and ${\tilde{G}}_{s}$ shape. As a consequence, the refocusing pattern is changed and the refocusing accuracy may be reduced for highly varying $G_{s}$ shapes. The GIRF alterations can be iteratively corrected for a given RF and $G_{s}$ shape^33^ or reduced limiting the $G_{s}$ frequency content.^17^ These methods, however, do not guarantee compliance with respect to the hardware constraints. Alternatively, the presented direct inclusion of the measured GIRF in the optimization compensated for gradient imperfections and resulted in a matched RF pulse that is linked to the filtered slice selective gradient shape ${\tilde{G}}_{s}$. Moreover, the unfiltered $G_{s}$ shapes are designed to exactly fulfill the prescribed hardware constraints to avoid any additional rescaling. We did not face any stability problems related to the use of an inverted GIRF in any of our optimization runs. The experiments show that this is beneficial for the practical performance of highly modulated slice selective gradient shapes in the presence of a limited gradient bandwidth.

To avoid additional known RF distortions associated with rapidly varying complex‐valued RF pulses^32^ we designed real valued RF pulses. In addition, we used the minimal gradient raster time of the MR system used for experimental validation (τ = 6.4 ms) to define both $G_{s}$ and RF waveform. Since the RF amplifier system is expected to have a much higher bandwidth compared to the gradient system, we did not expect significant alteration of the optimized RF shapes. We also assumed an idealized RF system for all experiments. It would be straightforward to include LTI RF system imperfections into the optimization framework analogously to the GIRF model, or to include additional slew rate constraints on the RF amplitude. For all our experiments we utilized the existing vendor RF non‐linearity correction prescan, which updated RF waveforms on a per scan basis. The residual imperfections in the RF waveforms were not directly measured, however, recently developed methods could be employed for this purpose and utilized to further refine the pre‐compensation of RF waveforms.^34^, ^35^

The optimization of all shown examples started with PINS^18^ RF pulses and blipped $G_{s}$ shapes that fulfill all given constraints. The use of PINS initials proved robust at yielding short pulse candidates for a large variation of parameters. This is in good accordance with our previous work on the design of SMS refocusing pulses without GIRF correction.^22^

Two representative cases (MB = 4 and MB = 5) were investigated in more detail. The low‐pass character and the frequency dependent phase delay of $H_{z}$ resulted in a smoothed and partly time‐shifted slice selective gradient shape. These alterations changed the temporal alignment with the optimized RF shape and resulted in degraded slice profiles, especially for the slices farther away from the isocenter. In contrast, there was a much smaller discrepancy in the refocusing profile closer to the isocenter. These alterations, however, could be corrected with the proposed $H_{z}$ GIRF incorporation during the optimization. Interestingly, the unfiltered $G_{s}$ shapes optimized with the ideal and measured GIRF had a similar visual appearance and a comparable frequency range. The still high slice selective gradient fluctuations come with the disadvantage of an increased gradient demand and mechanical vibrations. However, we did not face any problems with peripheral nerve stimulation in any of our experimental scans.

The inclusion of $H_{z}$ created a pre‐distorted slice selective gradient shape that compensates the GIRF low‐pass and phase characteristics and removed the undesired slice profile degradations.

This clear advantage comes with a price of slightly increased minimal pulse durations and SAR estimates. It should be noted, that the optimized global head SAR estimate is far from the used global head SAR constraint of 3.2 W/kg. The unconstrained phase spread problem has more freedom and therefore more local solutions with a shorter pulse duration. However, there is only a minor increase in the pulse duration when adding explicit phase spread constraints to each refocusing slice. Moreover, the combination of explicit phase constraints and $H_{z}$ resulted in only a slightly longer pulse duration with a vast reduction compared to the initial guess.

The robustness of the $H_{z}$ filtered initial guess and optimized pulses (MB = 4) with respect to time invariant and static $B_{0}$ and $B_{1}$ variations were investigated after the iterative design process with numerical Bloch simulations, see Figure 3. The reduced durations of the optimized pulses lead to lower slice displacement, but a higher $B_{0}$ sensitivity as a result of the variable k‐space velocity. The $H_{z}$ optimized and $H_{z}$ filtered results provide stable refocusing profiles in the range of ±150 Hz, which is in good agreement with time optimal results that assumed an idealized GIRF.^22^ Depending on the application more robust pulses may be required. Therefore, an inclusion of $B_{0}$/$B_{1}$ robustness into the optimization framework will be focus of future work.

The different gradient coil design of longitudinal (*z*‐direction) and transversal (*y*‐direction) leads to a slightly different GIRF for each direction. To achieve the best accuracy, the GIRF used in the optimization should fit with the gradient direction of the MR experiment. Nevertheless, there are only small differences between $H_{y}$ and $H_{z}$, see Figure 1. This directly translated to the numerical Bloch simulations using a different GIRF for optimization and simulation with only small slice profile deviations, see Figure 5. If the gradient direction is unknown prior to optimization, an averaged GIRF of different gradient directions could be used. This intermediate case results in smaller maximal slice profile deviations compared to use of a single distinct GIRF in the optimization and evaluation.

For validation we have performed 2 different experiments on 2 different MR systems of the same model. The phantom experiments clearly show the beneficial effects of the incorporated GIRF correction. Because of software limitations we used the GIRF measured from 1 MR system to correct pulses applied to another. The in vivo demonstration was further limited to gradient echo measurements. To comply with this restriction we used 9x‐downscaled refocusing pulses and applied them as excitation pulses. The performance of the scaled pulses was estimated with Bloch simulations in the excitation regime ($|2ab^{*}|$). Although the pulses were designed with respect to the crushed refocusing profile ($\left|b^{2}\right|$) it is reasonable to expect clean excitation slice profiles. Moreover, distorted slice profiles are recovered using the proposed design method. This is in good accordance with the observed in vivo results acquired on system 2. The good experimental performance implies that the gradient systems do have similar frequency responses, which we have observed before.^17^ However, more work is needed to understand the limits of using generic GIRFs to improve the applicability of the proposed method.

The proposed GIRF compensated design method was tested for various parameters including different MB factors (3−8), TBWP (2−4), THK (1−5 mm), FOV (90−210 mm), phase angle constraints and gradient directions.

In addition to the uniform signal strength, the GIRF‐corrected pulse design results in an additional reduction of residual slices outside the FOV. This reduction of aliased slices is in agreement with previously published work.^17^

The proposed method provides a new approach for GIRF‐corrected RF pulse design. The incorporation of the GIRF into the optimization framework generates a pre‐emphasized $G_{s}$ shape and a matched RF pulse that can be applied to further push the limits of SMS sequences such as clinically important turbo spin echo (TSE) based sequences,^36^ functional imaging^37^ or short SE diffusion applications.^38^

## 6 CONCLUSIONS

The inclusion of a hardware specific GIRF into the optimal control framework provides compensation of limited gradient performance and yields distinct refocusing slices, even for minimum duration RF and rapidly changing Gs shapes.

## Supporting information


**FIGURE S1** Comparison of optimized results with different MB factors (3−7) with fixed THK = 2 mm, TBWP = 4 and FOV = 120 mm. Row 1 shows the optimized slice selective gradient shapes before ($G_{s}$) and after convolution with the GIRF (${{\tilde{G}}_{s}}^{H_{z}}$). Row 2 shows an enlargement of 4 mm of the simulated refocusing profiles ${\left|b\left(z\right)\right|}^{2}$ and row 3 shows the phase angle $\arg(b_{N_{t}}{\left(z\right)}^{2})$ of each slice (range of 4 mm) after GIRF convolution
**FIGURE S2** Comparison of optimized results with different TBWP factors (2.5‐3.5) with fixed MB = 3, THK = 2 mm and FOV = 120 mm. Row 1 shows the optimized slice selective gradient shapes before ($G_{s}$) and after convolution with the GIRF (${{\tilde{G}}_{s}}^{H_{z}}$). Row 2 shows an enlargement of 4 mm of the simulated refocusing profiles ${\left|b\left(z\right)\right|}^{2}$ and row 3 shows the phase angle $\arg(b_{N_{t}}{\left(z\right)}^{2})$ of each slice (range of 4 mm) after GIRF convolution
**FIGURE S3** Comparison of optimized results with different THK (5‐1 mm) with fixed MB = 5, TBWP = 4 and FOV = 120 mm. Row 1 shows the optimized slice selective gradient shapes before ($G_{s}$) and after convolution with the GIRF (${{\tilde{G}}_{s}}^{H_{z}}$). Row 2 shows an enlargement of 8 mm of the simulated refocusing profiles ${\left|b\left(z\right)\right|}^{2}$ and row 3 shows the phase angle $\arg(b_{N_{t}}{\left(z\right)}^{2})$ of each slice (range of 8 mm) after GIRF convolution
**FIGURE S4** Comparison of optimized results with different field of view (FOV = 90‐210 mm) with fixed MB = 3, TBWP = 4 and THK = 2 mm. Row 1 shows the optimized slice selective gradient shapes before ($G_{s}$) and after convolution with the GIRF (${{\tilde{G}}_{s}}^{H_{z}}$). Row 2 shows an enlargement of 4 mm of the simulated refocusing profiles ${\left|b\left(z\right)\right|}^{2}$ and row 3 shows the phase angle $\arg(b_{N_{t}}{\left(z\right)}^{2})$ of each slice (range of 4 mm) after GIRF convolution
**FIGURE S5** Simulated excitation profiles of 9x‐downscaled $H_{i}$ and $H_{z}$ optimized refocusing pulses, shown in Figure 2. The excitation profiles are depicted in terms of the flip angle $\sin^{-1}(|2a_{N_{t}}\left(z\right)b_{N_{t}}{\left(z\right)}^{*}\left|\right)$. Note that despite the pulses have been optimized with respect to the refocusing profile $|b_{N_{t}}{\left(z\right)|}^{2}$ the 9x‐downscaled pulses result in clean slice profiles with a $20^{\circ }$ flip angle. The pulses are evaluated using the $H_{z}$ GIRF (scanner 1) filtered slice selective gradient ${{\tilde{G}}_{s}}^{H_{z}}$. The $H_{z}$ optimized pulse recovers the lower signal of the outer slices which is in accordance with the observed in‐vivo results in Figure 8
**TABLE S1** Performance of optimized SMS refocusing pulses (MB = 5, TBWP = 4, THK = 2 mm and FOV = 120 mm) for different GIRF directions ($H_{y}$, $H_{z}$ and $H_{\mathrm{yz}}$) with and without constraints on the phase angle of the refocusing profiles. All pulses are evaluated using the $H_{y}$ and $H_{\mathrm{yz}}$ GIRF filtered slice selective gradient ${{\tilde{G}}_{s}}^{H_{z}}$ and ${{\tilde{G}}_{s}}^{H_{\mathrm{yz}}}$ to analyze the influence of the GIRF direction. Depicted are the GIRF used in the optimization, maximal refocusing profile ($e_{s}$) and phase ($e_{p}$) errors
**TABLE S2** Performance of initial and optimized results with varying TBWP = 2‐4, MB= 3‐8 factor, THK = 1‐5 mm and FOV = 90‐210 mm. The optimization is done with the measured GIRF along the *z*‐direction ($H_{z}$) with explicit phase constraints. The slice and phase errors are evaluated after Bloch simulation with the GIRF filtered slice selective gradient shape ${{\tilde{G}}_{s}}^{H_{z}}$. Parameters used: maximal refocusing slice ($e_{s}$) and phase ($e_{p}$) errors, the SAR estimate ($SAR_{e}$) and the overall pulse duration (*T*)

## References

[1] Tsao J . Ultrafast imaging: principles, pitfalls, solutions, and applications. J Magn Reson Imaging. 2010;32:252–266. 20677249 10.1002/jmri.22239

[2] Blaimer M , Breuer F , Mueller M , Heidemann RM , Griswold MA , Jakob PM . SMASH, SENSE, PILS, GRAPPA: how to choose the optimal method. Topics Magn Reson Imaging. 2004;15:223–236. 10.1097/01.rmr.0000136558.09801.dd 15548953

[3] Deshmane A , Gulani V , Griswold MA , Seiberlich N . Parallel MR imaging. J Magn Reson Imaging. 2012;36:55–72. 22696125 10.1002/jmri.23639 PMC4459721

[4] Feinberg DA , Setsompop K . Ultra‐fast MRI of the human brain with simultaneous multi‐slice imaging. J Magn Reson. 2013;229:90–100. 23473893 10.1016/j.jmr.2013.02.002 PMC3793016

[5] Barth M , Breuer F , Koopmans P , Norris D , Poser B . Simultaneous multislice (SMS) imaging techniques. Magn Reson Med. 2016;75:63–81. 26308571 10.1002/mrm.25897 PMC4915494

[6] Mueller S . Multifrequency selective RF pulses for multislice MR imaging. Magn Reson Med. 1988;6:364–371. 3362070 10.1002/mrm.1910060315

[7] Norris DG , Boyacioglu R , Schulz J , Barth M , Koopmans PJ . Application of PINS radiofrequency pulses to reduce power deposition in RARE/turbo spin echo imaging of the human head. Magn Reson Med. 2014;71:44–49. 24150771 10.1002/mrm.24991

[8] Grissom WA , Setsompop K , Hurley SA , Tsao J , Velikina JV , Samsonov AA . Advancing RF pulse design using an open‐competition format: report from the 2015 ISMRM challenge. Magn Reson Med. 2017;78:1352–1361. 27790754 10.1002/mrm.26512 PMC5408273

[9] Wong E . Optimized phase schedules for minimizing peak RF power in simultaneous multi‐slice RF excitation pulses. In Proceedings of the International Society for Magnetic Resonance in Medicine, Melbourne, Australia, 2012. Abstract #2209.

[10] Auerbach EJ , Xu J , Yacoub E , Moeller S , Uğurbil K . Multiband accelerated spin‐echo echo planar imaging with reduced peak RF power using time‐shifted RF pulses. Magn Reson Med. 2013;69:1261–1267. 23468087 10.1002/mrm.24719 PMC3769699

[11] Sharma A , Lustig M , Grissom WA . Root‐flipped multiband refocusing pulses. Magn Reson Med. 2016;75:227–237. 25704154 10.1002/mrm.25629 PMC4546574

[12] Conolly S , Nishimura D , Macovski A , Glover G . Variable‐rate selective excitation. J Magn Reson. 1988;78:440–458.

[13] Hargreaves BA , Cunningham CH , Nishimura DG , Conolly SM . Variable‐rate selective excitation for rapid MRI sequences. Magn Reson Med. 2004;52:590–597. 15334579 10.1002/mrm.20168

[14] Setsompop K , Gagoski BA , Polimeni JR , Witzel T , Wedeen VJ , Wald LL . Blipped‐controlled aliasing in parallel imaging for simultaneous multislice echo planar imaging with reduced g‐factor penalty. Magn Reson Med. 2012;67:1210–1224. 21858868 10.1002/mrm.23097 PMC3323676

[15] Grissom WA , McKinnon GC , Vogel MW . Nonuniform and multidimensional Shinnar‐Le Roux RF pulse design method. Magn Reson Med. 2012;68:690–702. 22161690 10.1002/mrm.23269

[16] Kobayashi N , Ugurbil K , Wu X . Shortening nonlinear phase multiband refocusing pulses with VERSE. In Proceedings of the International Society for Magnetic Resonance in Medicine, Singapore, Republic of Singapore, 2016. Abstract #3253.

[17] Abo Seada S , Price AN , Schneider T , Hajnal JV , Malik SJ . Multiband RF pulse design for realistic gradient performance. Magn Reson Med. 2019;81:362–376. 30277267 10.1002/mrm.27411 PMC6334175

[18] Norris DG , Koopmans PJ , Boyacioğlu R , Barth M . Power independent of number of slices (PINS) radiofrequency pulses for low‐power simultaneous multislice excitation. Magn Reson Med. 2011;66:1234–1240. 22009706 10.1002/mrm.23152

[19] Eichner C , Wald LL , Setsompop K . A low power radiofrequency pulse for simultaneous multislice excitation and refocusing. Magn Reson Med. 2014;72:949–958. 25103999 10.1002/mrm.25389

[20] Aigner CS , Clason C , Rund A , Stollberger R . Efficient high‐resolution RF pulse design applied to simultaneous multi‐slice excitation. J Magn Reson. 2016;263:33–44. 26773524 10.1016/j.jmr.2015.11.013

[21] Rund A , Aigner CS , Kunisch K , Stollberger R . Magnetic resonance RF pulse design by optimal control with physical constraints. IEEE Trans Med Imaging. 2018;37:461–472. 28981407 10.1109/TMI.2017.2758391

[22] Rund A , Aigner CS , Kunisch K , Stollberger R . Simultaneous multislice refocusing via time optimal control. Magn Reson Med. 2018;80:1416–1428. 29427294 10.1002/mrm.27124

[23] Vannesjo SJ , Haeberlin M , Kasper L , et al. Gradient system characterization by impulse response measurements with a dynamic field camera. Magn Reson Med. 2013;69:583–593. 22499483 10.1002/mrm.24263

[24] Campbell‐Washburn AE , Xue H , Lederman RJ , Faranesh AZ , Hansen MS . Real‐time distortion correction of spiral and echo planar images using the gradient system impulse response function. Magn Reson Med. 2016;75:2278–2285. 26114951 10.1002/mrm.25788 PMC4691439

[25] Cavusoglu M , Mooiweer R , Pruessmann KP , Malik SJ . VERSE‐guided parallel RF excitations using dynamic field correction. NMR Biomed. 2017;30:e3697. 10.1002/nbm.3697 PMC5484370 28211968

[26] Harkins KD , Does MD , Grissom WA . Iterative method for predistortion of MRI gradient waveforms. IEEE Trans Med Imaging. 2014;33:1641–1647. 24801945 10.1109/TMI.2014.2320987 PMC4128553

[27] Busch J , Vannesjo SJ , Barmet C , Pruessmann KP , Kozerke S . Analysis of temperature dependence of background phase errors in phase‐contrast cardiovascular magnetic resonance. J Cardiovasc Magn Reson. 2014;16:1–12. 25497004 10.1186/s12968-014-0097-6 PMC4263200

[28] Jaynes E . Matrix treatment of nuclear induction. Phys Rev. 1955;98:1099–1105.

[29] Pauly J , Le Roux P , Nishimura D , Macovski A . Parameter relations for the Shinnar–Le Roux selective excitation pulse design algorithm. IEEE Trans Med Imaging. 1991;10:53–65. 18222800 10.1109/42.75611

[30] Vernickel P , Röschmann P , Findeklee C , et al. Eight‐channel transmit/receive body MRI coil at 3T. Magn Reson Med. 2007;58:381–389. 17654592 10.1002/mrm.21294

[31] Papadakis NG , Wilkinson AA , Carpenter T , Hall LD . A general method for measurement of the time integral of variant magnetic field gradients: application to 2D spiral imaging. Magn Reson Imaging. 1997;15:567–578. 9254001 10.1016/s0730-725x(97)00014-3

[32] Abo Seada S , Price AN , Hajnal JV , Malik SJ . Optimized amplitude modulated multiband RF pulse design. Magn Reson Med. 2017;78:2185–2193. 28097733 10.1002/mrm.26610 PMC5697703

[33] Abo Seada S , Hajnal JV , Malik SJ . A simple optimisation approach to making time efficient verse‐multiband pulses feasible on non‐ideal gradients. In Proceedings of the 25th Annual Meeting of ISMRM, Honolulu, HI, 2017. Abstract #5049.

[34] Landes VL , Nayak KS . Simple method for RF pulse measurement using gradient reversal. Magn Reson Med. 2018;79:2642–2651. 28905516 10.1002/mrm.26920 PMC5821550

[35] Landes VL , Nayak KS . Improved multi‐band RF performance using GRATER‐based predistortion. In 26th Annual Meeting of ISMRM, France, 2018. Abstract #0171.

[36] Gagoski BA , Bilgic B , Eichner C , et al. RARE/turbo spin echo imaging with simultaneous multislice Wave‐CAIPI. Magn Reson Med. 2015;73:929–938. 25640187 10.1002/mrm.25615 PMC4334698

[37] Chen L , Vu AT , Xu J , et al. Evaluation of highly accelerated simultaneous multi‐slice EPI for fMRI. NeuroImage. 2015;104:452–459. 25462696 10.1016/j.neuroimage.2014.10.027 PMC4467797

[38] Setsompop K , Fan Q , Stockmann J , et al. High‐resolution in vivo diffusion imaging of the human brain with generalized slice dithered enhanced resolution: simultaneous multislice (gSlider‐SMS). Magn Reson Med. 2018;79:141–151. 28261904 10.1002/mrm.26653 PMC5585027

