# Supplementary material for: Time optimal control‐based RF pulse design under gradient imperfections
Source: Magn Reson Med. 2019 Aug 23;83(2):561–74. doi: 10.1002/mrm.27955 (PMC6899978; doi:10.1002/mrm.27955)
Supplement: Supplementary file 1 — FIGURE S1 Comparison of optimized results with different MB factors (3−7) with fixed THK = 2 mm, TBWP = 4 and FOV = 120 mm. Row 1 shows the optimized slice selective gradient shapes before (Gs) and after convolution with the GIRF (G~sHz). Row 2 shows an enlargement of 4 mm of the simulated refocusing profiles |b(z)|2 and row 3 shows the phase angle arg(bNt(z)2) of each slice (range of 4 mm) after GIRF convolution FIGURE S2 Comparison of optimized results with different TBWP factors (2.5‐3.5) with fixed MB = 3, THK = 2 mm and FOV = 120 mm. Row 1 shows the optimized slice selective gradient shapes before (Gs) and after convolution with the GIRF (G~sHz). Row 2 shows an enlargement of 4 mm of the simulated refocusing profiles |b(z)|2 and row 3 shows the phase angle arg(bNt(z)2) of each slice (range of 4 mm) after GIRF convolution FIGURE S3 Comparison of optimized results with different THK (5‐1 mm) with fixed MB = 5, TBWP = 4 and FOV = 120 mm. Row 1 shows the optimized slice selective gradient shapes before (Gs) and after convolution with the GIRF (G~sHz). Row 2 shows an enlargement of 8 mm of the simulated refocusing profiles |b(z)|2 and row 3 shows the phase angle arg(bNt(z)2) of each slice (range of 8 mm) after GIRF convolution FIGURE S4 Comparison of optimized results with different field of view (FOV = 90‐210 mm) with fixed MB = 3, TBWP = 4 and THK = 2 mm. Row 1 shows the optimized slice selective gradient shapes before (Gs) and after convolution with the GIRF (G~sHz). Row 2 shows an enlargement of 4 mm of the simulated refocusing profiles |b(z)|2 and row 3 shows the phase angle arg(bNt(z)2) of each slice (range of 4 mm) after GIRF convolution FIGURE S5 Simulated excitation profiles of 9x‐downscaled Hi and Hz optimized refocusing pulses, shown in Figure 2. The excitation profiles are depicted in terms of the flip angle sin-1(|2aNt(z)bNt(z)*|). Note that despite the pulses have been optimized with respect to the refocusing profile |bNt(z)|2 the 9x‐downscaled pulses [file MRM-83-561-s001.pdf]

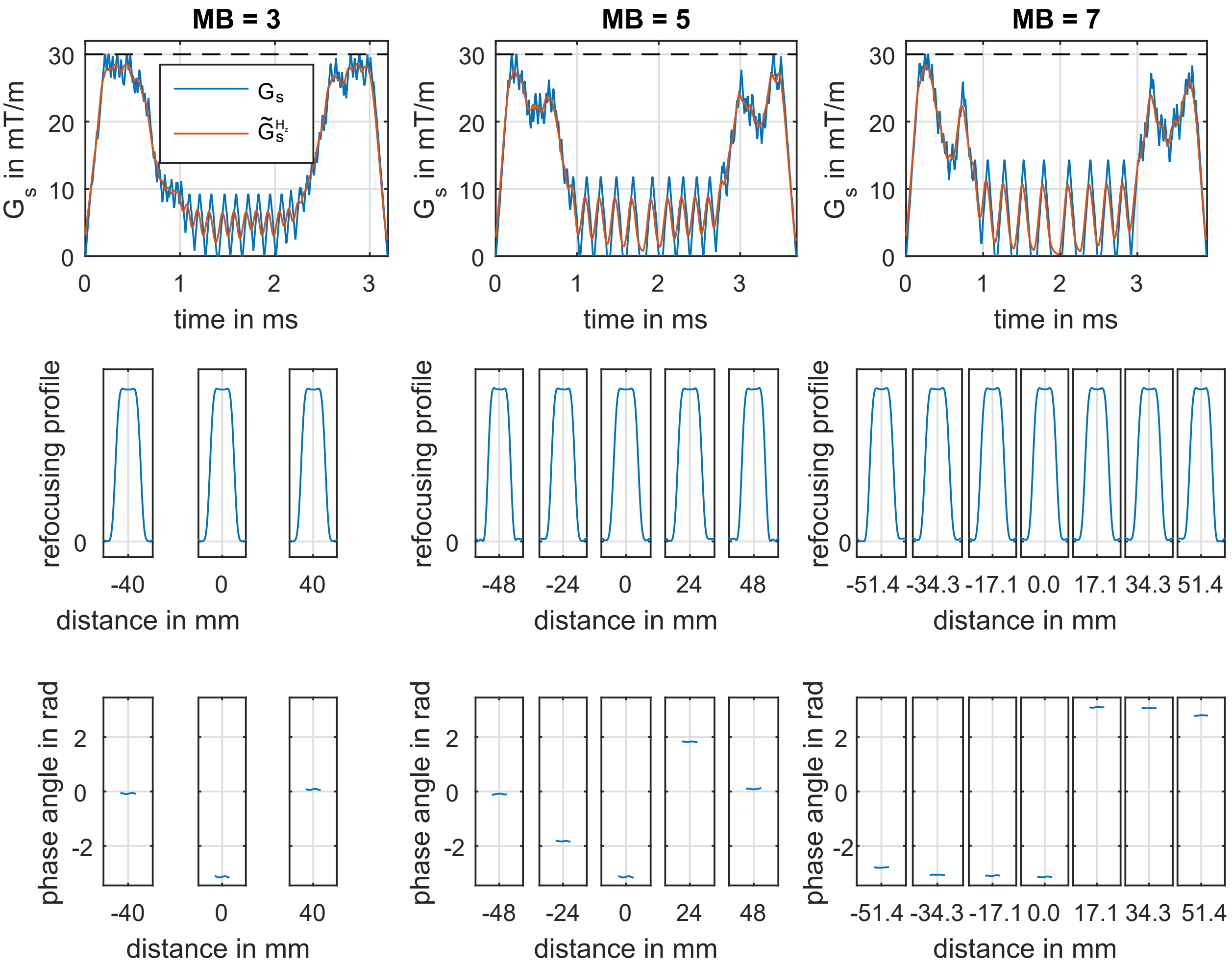

Supporting Information Figure S 1: Comparison of optimized results with different MB factors $(3 - 7)$ with fixed THK $= 2$ mm, TBWP $= 4$ and FOV $= 120$ mm. Row 1 shows the optimized slice selective gradient shapes before (G_s) and after convolution with the GIRF $(\tilde{G}_s^{H_z})$. Row 2 shows an enlargement of 4 mm of the simulated refocusing profiles $|b_{N_t}(z)|^2$ and row 3 shows the phase angle $\arg(b_{N_t}(z)^2)$ of each slice (range of 4 mm) after GIRF convolution.

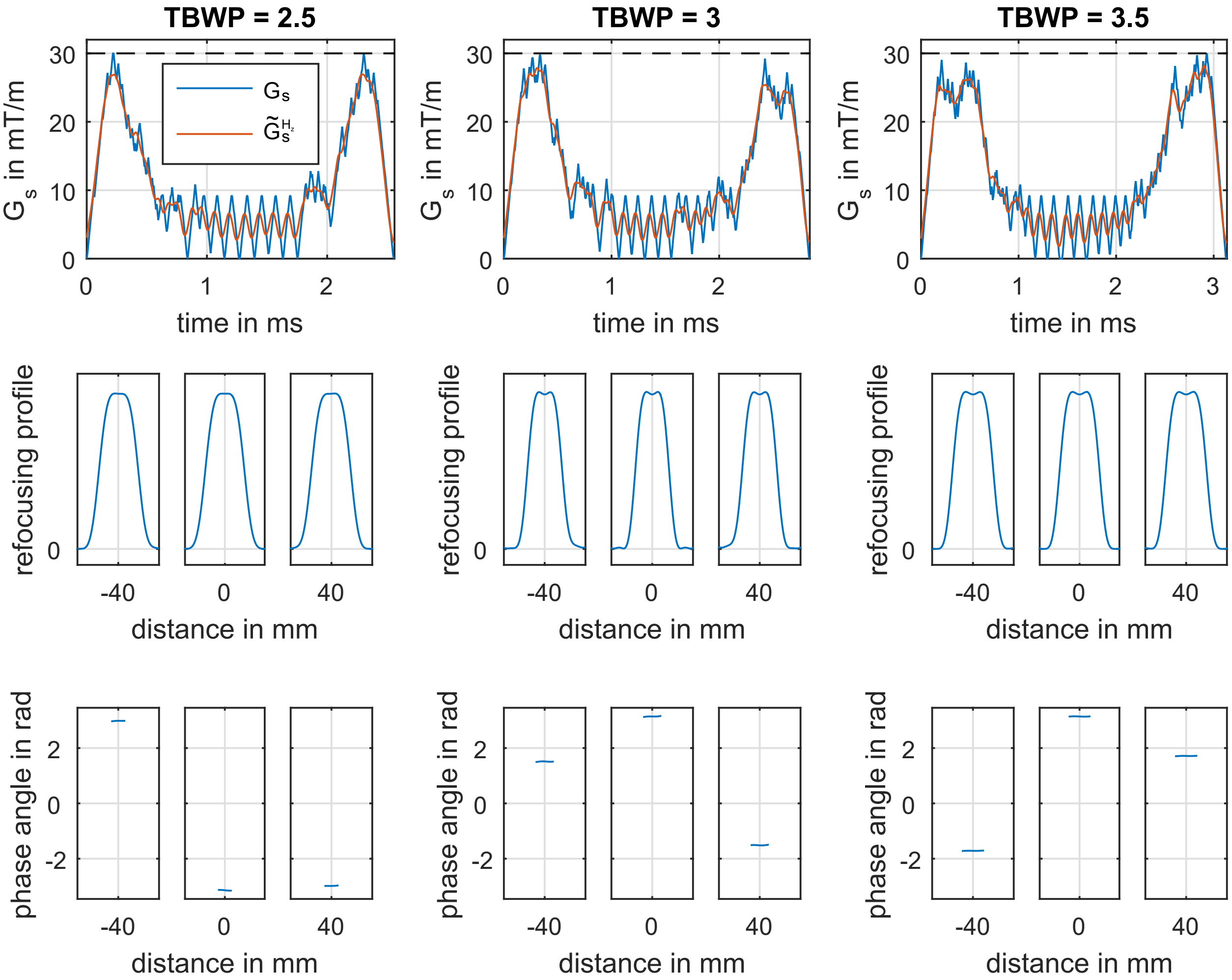

Supporting Information Figure S2: Comparison of optimized results with different TBWP factors $(2.5 - 3.5)$ with fixed MB $= 3$, THK $= 2$ mm and FOV $= 120$ mm. Row 1 shows the optimized slice selective gradient shapes before (G_s) and after convolution with the GIRF $(\tilde{G}_{\mathrm{s}}^{H_z})$. Row 2 shows an enlargement of 4 mm of the simulated refocusing profiles $|b_{N_t}(z)|^2$ and row 3 shows the phase angle $\arg(b_{N_t}(z)^2)$ of each slice (range of 4 mm) after GIRF convolution.

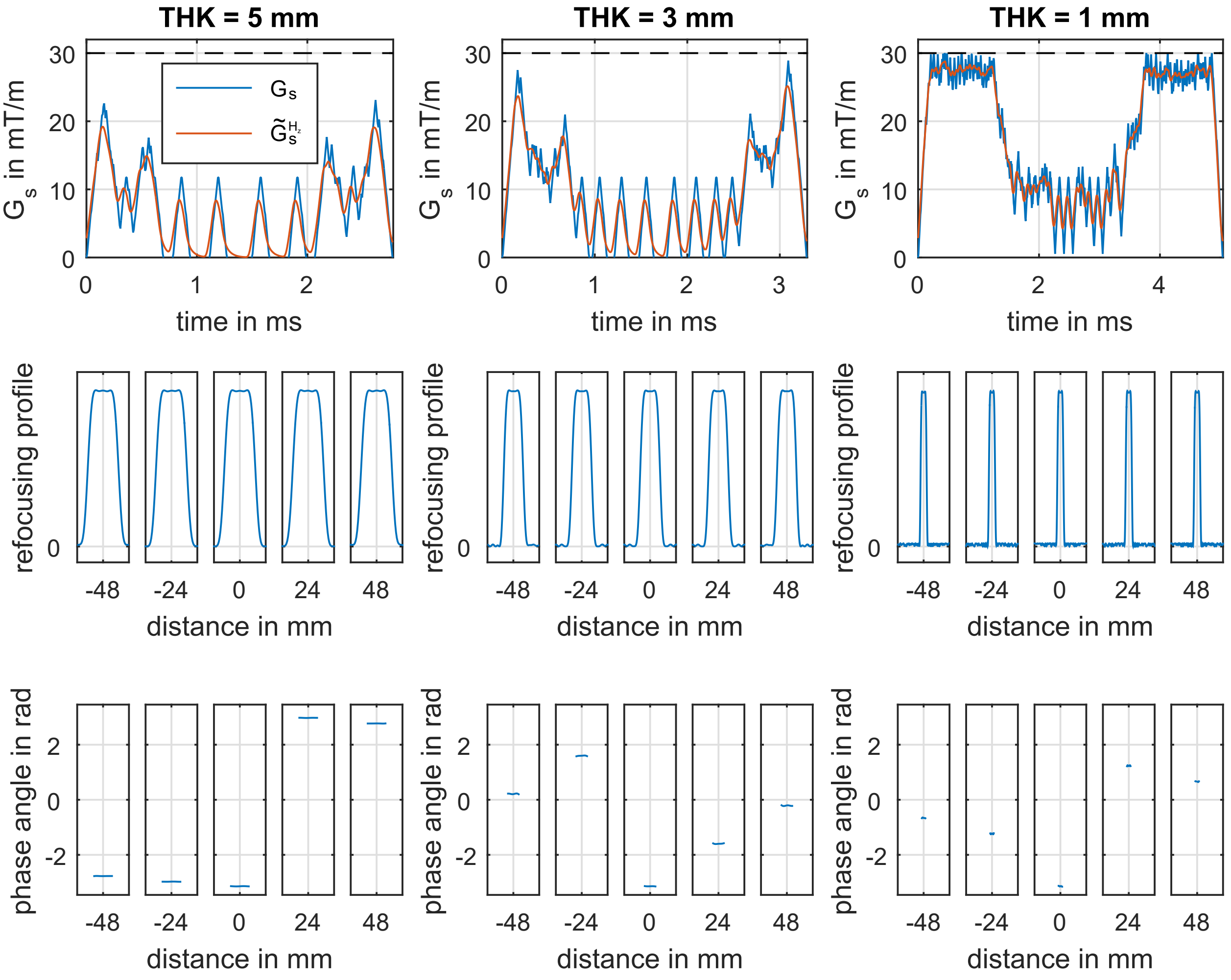

Supporting Information Figure S3: Comparison of optimized results with different THK (5 − 1 mm) with fixed MB = 5, TBWP = 4 and FOV = 120 mm. Row 1 shows the optimized slice selective gradient shapes before (G_s) and after convolution with the GIRF ($\tilde{G}_s^{H_z}$). Row 2 shows an enlargement of 8 mm of the simulated refocusing profiles $|b_{N_t}(z)|^2$ and row 3 shows the phase angle $\arg(b_{N_t}(z)^2)$ of each slice (range of 8 mm) after GIRF convolution.

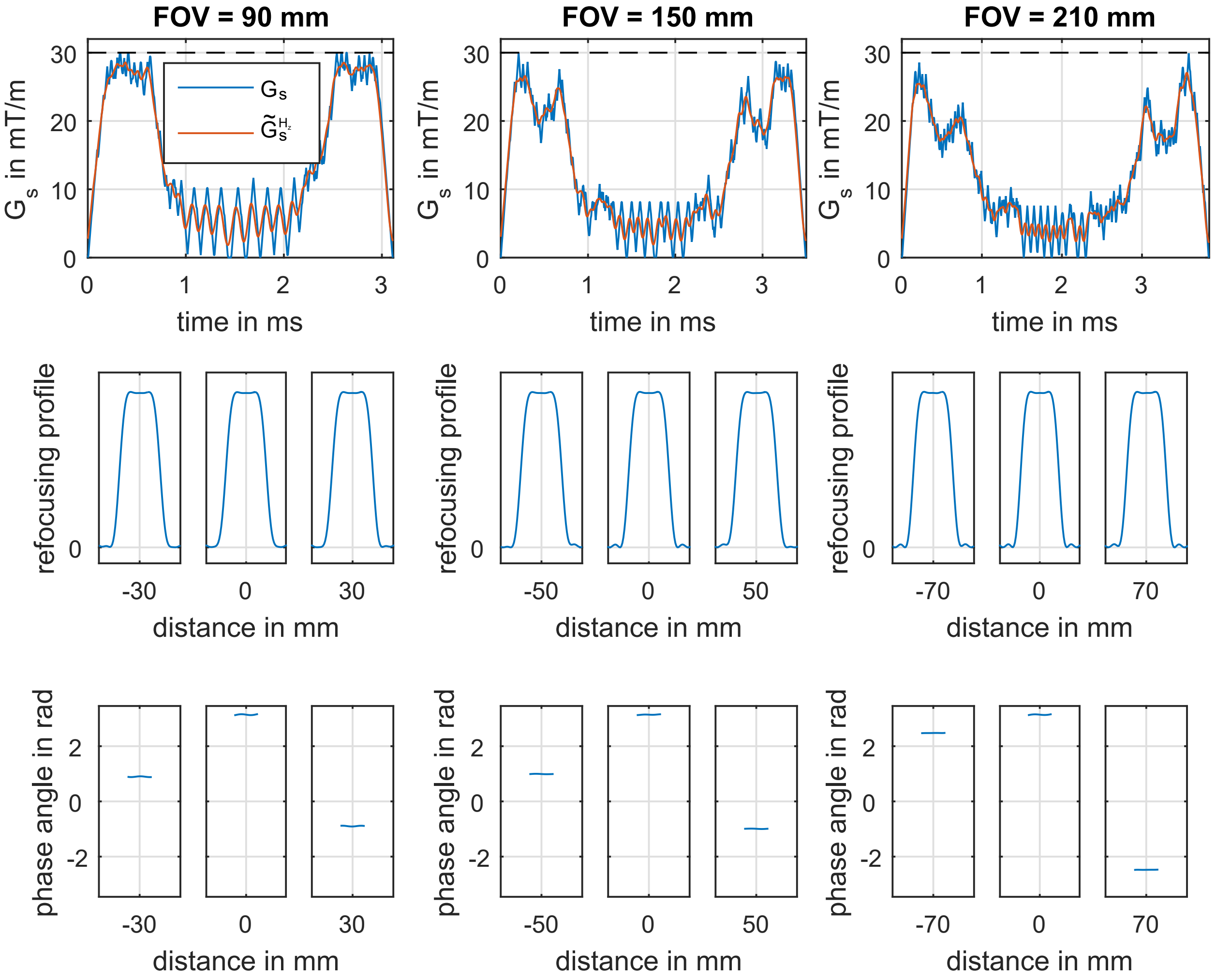

Supporting Information Figure S4: Comparison of optimized results with different field of view (FOV = 90 – 210 mm) with fixed MB = 3, TBWP = 4 and THK = 2 mm. Row 1 shows the optimized slice selective gradient shapes before (G_s) and after convolution with the GIRF ($\tilde{G}_s^{H_z}$). Row 2 shows an enlargement of 4 mm of the simulated refocusing profiles $|b_{N_t}(z)|^2$ and row 3 shows the phase angle $\arg(b_{N_t}(z)^2)$ of each slice (range of 4 mm) after GIRF convolution.

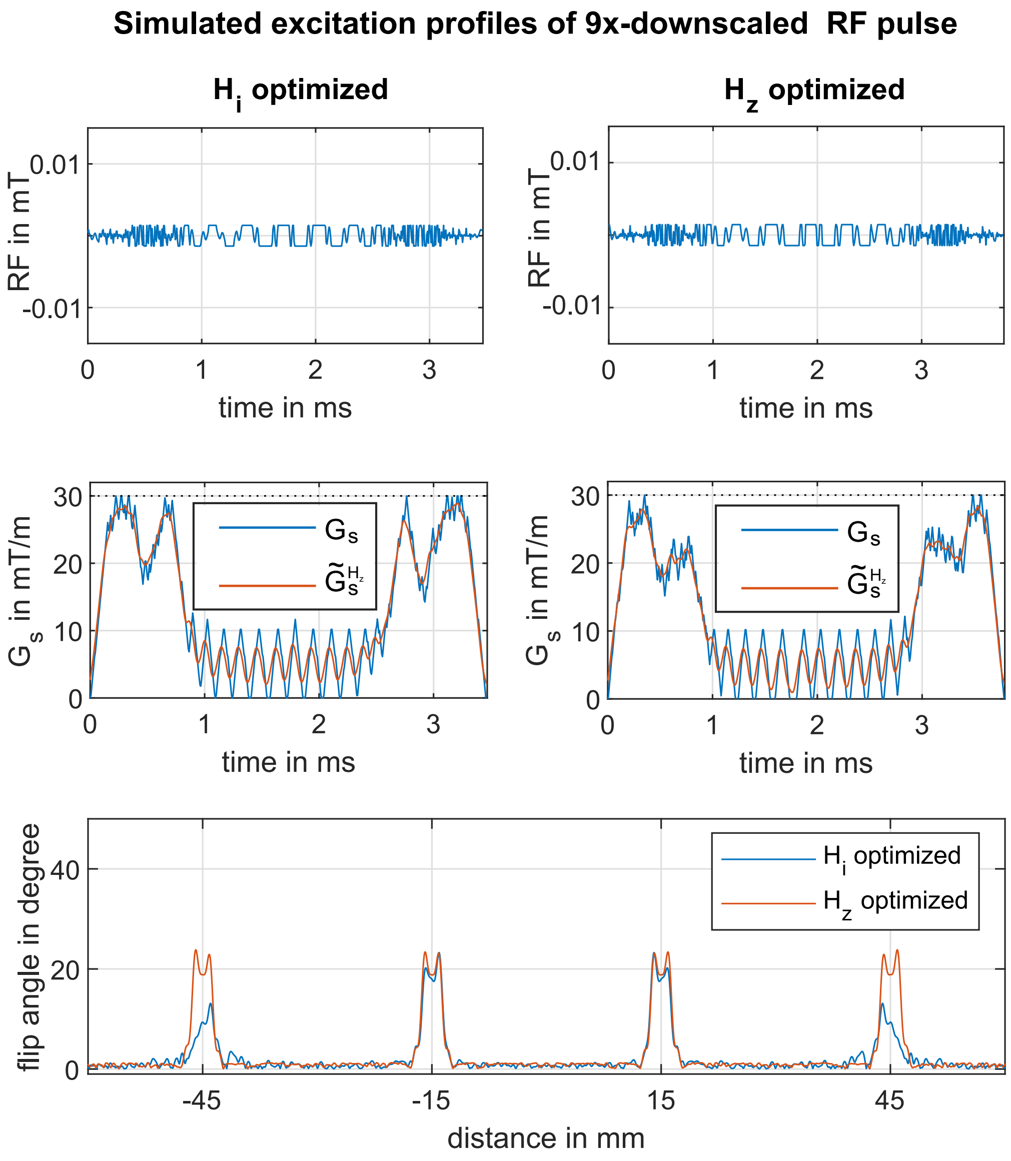

Supporting Information Figure S5: Simulated excitation profiles of 9x-downscaled H_i and H_z optimized refocusing pulses, shown in Figure 2. The excitation profiles are depicted in terms of the flip angle $sin^{-1}(|2a_{N_t}(z)b_{N_t}(z)^*|)$. Note that despite the pulses have been optimized with respect to the refocusing profile $|b_{N_t}(z)|^2$ the 9x-downscaled pulses result in clean slice profiles with a 20° flip angle. The pulses are evaluated using the H_z GIRF (scanner 1) filtered slice selective gradient $\tilde{G}_\mathrm{s}^{H_z}$. The H_z optimized pulse recovers the lower signal of the outer slices which is in accordance with the observed in-vivo results in Figure 8.

Supporting Information Table S1: Performance of optimized SMS refocusing pulses (MB = 5, TBWP = 4, THK = 2 mm and FOV = 120 mm) for different GIRF directions (H_y, H_z and H_{yz}) without and with constraints on the phase angle of the refocusing profiles. All pulses are evaluated using the H_y and H_{yz} GIRF filtered slice selective gradient $\tilde{G}_{\mathrm{s}}^{H_z}$ and $\tilde{G}_{\mathrm{s}}^{H_{yz}}$ to analyze the influence of the GIRF direction. Depicted are the GIRF used in the optimization, maximal refocusing profile (e_s) and phase (e_p) errors.

		evaluated with H_y		evaluated with H_{yz}	
		max $\lvert e_s \rvert$ a.u.	max $\lvert e_p \rvert$ rad	max $\lvert e_s \rvert$ a.u.	max $\lvert e_p \rvert$ rad
without phase constraints	H_i optimized	0.883	0.867	0.915	0.943
	H_y optimized	0.021	1.531	0.079	1.527
	H_z optimized	0.256	2.259	0.102	2.261
	H_{yz} optimized	0.082	0.689	0.020	0.706
with phase constraints	H_i optimized	0.902	0.819	0.911	0.734
	H_y optimized	0.021	0.024	0.063	0.034
	H_z optimized	0.194	0.054	0.083	0.028
	H_{yz} optimized	0.086	0.053	0.020	0.017

Supporting Information Table S2: Performance of initial and optimized results with varying TBWP = 2 − 4, MB = 3 − 8 factor, THK = 1 − 5 mm and FOV = 90 − 210 mm. The optimization is done with the measured GIRF along the z-direction (H_z) with explicit phase constraints. The slice and phase errors are evaluated after Bloch simulation with the GIRF filtered slice selective gradient shape $\tilde{G}_{\mathrm{s}}^{H_z}$. Parameters used: maximal refocusing slice (e_s) and phase (e_p) errors, the SAR estimate (SAR_e) and the overall pulse duration (T).

				initial		H_i optimized		H_z optimized	
MB	TBWP	THK	FOV	SAR_e	T	SAR_e	T	SAR_e	T
a.u.	a.u.	mm	mm	$\mathrm{W\,kg^{-1}}$	ms	$\mathrm{W\,kg^{-1}}$	ms	$\mathrm{W\,kg^{-1}}$	ms
3	2	2	120	0.48	7.48	1.14	1.95	1.23	2.16
3	2.5	2	120	0.56	11.62	1.18	2.32	1.34	2.56
3	3	2	120	0.56	13.15	1.27	2.60	1.41	2.84
3	3.5	2	120	0.58	15.32	1.30	2.85	1.49	3.14
3	4	2	120	0.60	18.54	1.42	3.01	1.59	3.19
4	4	2	120	0.61	17.41	1.65	3.19	1.66	3.51
5	4	2	120	0.61	16.14	1.65	3.42	1.86	3.69
6	4	2	120	0.61	15.30	1.74	3.46	1.91	3.82
7	4	2	120	0.61	14.59	1.78	3.49	1.98	3.90
8	4	2	120	0.62	14.08	1.82	3.65	1.94	4.15
5	4	1	120	0.57	24.67	1.75	4.98	1.97	5.04
5	4	2	120	0.61	16.14	1.84	3.29	1.86	3.69
5	4	3	120	0.61	13.35	1.35	2.94	1.67	3.30
5	4	4	120	0.61	11.83	1.20	2.75	1.51	2.98
5	4	5	120	0.62	10.99	1.25	2.48	1.40	2.78
3	4	2	90	0.60	17.41	1.33	2.92	1.52	3.12
3	4	2	120	0.60	18.54	1.42	3.01	1.59	3.19
3	4	2	150	0.59	19.92	1.46	3.08	1.59	3.50
3	4	2	180	0.58	22.34	1.53	3.16	1.54	3.74
3	4	2	210	0.61	22.45	1.48	3.21	1.66	3.84
